# Accelerated acquisition of carotid MR angiography using 3D gradient-echo imaging with two-point Dixon

**DOI:** 10.1007/s00234-020-02452-6

**Published:** 2020-05-18

**Authors:** Ryusuke Irie, Shiori Amemiya, Tsuyoshi Ueyama, Yuichi Suzuki, Kouhei Kamiya, Hidemasa Takao, Harushi Mori, Osamu Abe

**Affiliations:** 1 Department of Radiology, Graduate School of Medicine, The University of Tokyo, 7-3-1 Hongo, Bunkyo-ku, Tokyo, 113-8655 Japan; 2 Department of Radiology, The University of Tokyo Hospital, Tokyo, Japan; 3 Department of Radiology, School of Medicine, Jichi Medical University, Tochigi, Japan

**Keywords:** MR angiography, Two-point Dixon, Scan time

## Abstract

This pilot study tests the feasibility of rapid carotid MR angiography using the liver acquisition with volume acceleration-flex technique (LAVA MRA). Seven healthy volunteers and 21 consecutive patients suspected of carotid stenosis underwent LAVA and conventional time-of-flight (cTOF) MRAs. Artery-to-fat and artery-to-muscle signal intensity ratios were manually measured. LAVA MRA exhibited a significantly larger artery-to-fat signal intensity ratio compared with cTOF MRA in all slices (*P* < 0.001) and exhibited a larger (*P* < 0.001) or equivalent (*P* = 1.0) artery-to-muscle signal intensity ratio in the extracranial carotid arteries. The image quality of the cervical carotid bifurcation and the signal change on each MRA were visually assessed and compared among the MRAs. There was no significant difference between the two MRAs in visual assessment. LAVA MRA can provide visualization similar to cTOF MRA in the evaluation of the cervical carotid bifurcation while reducing scan time by one-fifth.

**Electronic supplementary material:**

The online version of this article (10.1007/s00234-020-02452-6) contains supplementary material, which is available to authorized users.

## Introduction

Three-dimensional (3D) time-of-flight MR angiography (TOF MRA) is a widely used non-contrast-enhanced (CE) procedure for assessing the carotid artery [1]. However, TOF MRA generally takes a few minutes to acquire and is consequently more susceptible to motion artifacts due to swallowing or gross body movement [2–4]. When imaging patients with acute symptoms or patients who have difficulty maintaining posture, image quality can be improved by reducing scan time.

In this study, to reduce the scan time of carotid inflow-dependent MRA, we applied the liver acquisition with volume acceleration-flex (LAVA-Flex) technique. LAVA-Flex is a 3D fast spoiled gradient-echo T1-weighted imaging technique that uses the two-point Dixon method [5]. In the LAVA-Flex technique, because the repetition time (TR) is set as short as one-sixth of the TR of the conventional TOF MRA (cTOF MRA), the scan time can be remarkably shortened.

The purposes of this study were to investigate optimal imaging conditions for clinical use of carotid MRA using LAVA-Flex (LAVA MRA) and to investigate whether LAVA MRA yields diagnostic images comparable with those of cTOF MRA in patients suspected of cervical carotid stenosis.

## Materials and methods

This prospective study was approved by the Institutional Review Board (2390-(9)). Seven healthy volunteers (M:F = 6:1, age 30.0 ± 4.7) and 21 consecutive patients suspected of cervical carotid stenosis (M:F = 14:7, age 73.0 ± 7.6) were included in this study. Written informed consent was obtained from all participants.

All subjects underwent both cTOF and LAVA MRAs for the cervical carotid artery. For cases with cervical carotid stenosis, a 3D fat-saturated T1 Cube sequence was additionally scanned. MRI scans were conducted using a 3.0-T clinical scanner (Signa Premier Ver27; GE Healthcare, Milwaukee, WI, USA) with a 21-ch head-neck coil. The center of the scanning area in the cranio-caudal direction was set at 1 cm above the carotid bifurcation. The imaging parameters for LAVA MRA were as follows: TR = 4.9 ms; TE = 1.1 ms (out-of-phase) and 2.2 ms (in-phase); FA = 4°, 7°, and 10°; bandwidth = 1302.1 Hz/pixel; field of view = 220 mm × 220 mm; matrix size = 256 × 224; parallel imaging acceleration factor = 2; slice orientation = transaxial plane; slice thickness = 2 mm × 50 slices (reconstruction 2 mm with 1 mm overlap); number of imaging slabs = 1; number of excitations = 1; scan time = 29 s. The spatial parameters, including the positioning and acceleration factor, of cTOF MRA were set the same as those for LAVA MRA. cTOF MRA was acquired using ramped excitation pulses with flow compensation, and without fat suppression. Other factors for cTOF MRA were as follows: TR = 30.0 ms; TE = 3.4 ms; FA = 20°; bandwidth = 244.1 Hz/pixel; scan time = 152 s. Venous inflow saturation was applied for both LAVA and cTOF MRAs. The imaging parameters for 3D fat-saturated T1 Cube were as follows: TR = 602 ms; TE = 11.9 ms; initial/minimum FA = 120°/25°; bandwidth = 488.3 Hz/pixel; field of view = 200 mm × 200 mm; matrix size = 256 × 256 (reconstruction 512 × 512); parallel imaging acceleration factors = 2.0 (phase), 1.5 (slice); compressed sensing factor = 1.1; slice orientation = coronal plane; slice thickness = 1 mm × 180 slices (reconstruction 0.5 mm); number of excitations = 1; scan time = 223 s.

In order to determine the optimal flip angle (FA) for the LAVA-Flex method, imaging was performed by changing the FA to 4°, 7°, 10°, and 13° in healthy volunteers.

One patient had left cervical internal carotid artery (ICA) occlusion, so region-of-interest (ROI) measurements were made on seven volunteers and 20 patients. The ROIs were placed in six slices of MRA source images. First, the slice of the carotid bifurcation was set as an index, and the slices 1 and 2 cm inferior and 1, 2, and 4 cm superior to the index slice were chosen for measurement. Six images were designated as slice 1 to slice 6, from bottom to top. For each slice of the cTOF MRA, ROIs were manually set to cover the maximum area within the bilateral carotid arteries while avoiding the plaque (approximately 5 mm in diameter). The similar or larger size ROIs were placed within the bilateral sternocleidomastoid muscles (or masseter muscles) and subcutaneous fat in the posterior neck to measure the average signal. To minimize arbitrariness, all ROIs were copied to the same position in the same level of slices for other MRAs. One radiologist (experience of 7 years) and two MR technological specialists (experience of 14 years and 15 years) created ROIs, and another radiologist (experience of 14 years) checked them (Supplement 1). For each of the six slices, the signal intensity of each structure was measured and artery-to-fat (SI_artery/fat_) and artery-to-muscle (SI_artery/muscle_) signal intensity ratios were calculated. The signal ratios measured on the left and right were averaged, and a comparison between the MRA sequences was performed for each slice.

For visual assessment, two board-certified radiologists (experience of 14 and 7 years) independently evaluated each MRA image and the final score of the visual assessment was reached by consensus. The image quality based on the shape of cervical carotid bifurcation was assessed visually with the maximum intensity projection (MIP) images ((1) nondiagnostic: the presence or absence of stenosis cannot be evaluated in some part of the bifurcation; (2) less diagnostic: partly difficult to evaluate; (3) diagnostic: easy to evaluate). The signal drop due to vortex flow at the cervical carotid bifurcation was assessed visually with the axial source images ((1) signal drop > 50% of the cross-sectional area; (2) signal drop < 50%; (3) signal drop < 25%; (4) no signal drop). The signal drop due to vortex flow was evaluated separately for the left and right, and for one patient with a left cervical ICA occlusion, only the right side was evaluated. Eighteen cervical carotid stenosis lesions were identified in 11 out of 21 patients. In patients with cervical carotid stenosis, the stenosis rate was measured using axial images of LAVA MRA with FA 10° and cTOF MRA according to the European Carotid Surgery Trial method [6]. We measured the diameter of patent lumen and outer diameter of ICA where stenosis was the most severe on axial images. We defined the stenosis rate over 25% was positive.

Statistical analysis was performed using one-way repeated-measures analysis of variance (ANOVA) with the Bonferroni method for post hoc comparisons in the ROI study, the Friedman test with the Nemenyi test for post hoc comparisons in the visual assessment, and paired *t* test in the evaluation of the cervical carotid stenosis rate. The inter-rater agreement was statistically determined by using Cohen’s weighted kappa analysis. All analyses were performed using SPSS 22 software (SPSS Inc., Chicago, IL) and JMP pro 14.1 (SAS Institute, Cary, NC, USA).

## Results

### Pilot study in normal volunteers

LAVA MRA was imaged with FA set at 4°, 7°, 10°, and 13° (Supplement 2). At a FA of 4°, the blood flow signal was weak and not suitable for evaluation. As the FA increased, the intravascular signal increased. However, at a FA of 13°, the signal drop at the bifurcation due to the vortex flow was noticeable, and the distal part of the vertebral artery was poorly rendered in the MIP image. Therefore, we set the FA to 7° and 10° for patient data acquisition (Table 1).

**Table 1:** Visual evaluation scores of the young healthy volunteers

	FA 4°	FA 7°	FA 10°	cTOF
Image quality of MIP images	1.00 ± 0.00	3.00 ± 0.00	3.00 ± 0.00	3.00 ± 0.00
Inhomogeneity due to the vortex flow	3.86 ± 0.36	2.29 ± 1.07	1.86 ± 0.53	1.86 ± 0.53

Data are mean ± standard deviation. *FA 4°*, flip angle 4° (LAVA); *FA 7°*, flip angle 7° (LAVA); *FA 10°*, flip angle 10° (LAVA); *cTOF*, conventional time-of-flight; *MIP*, maximum intensity projection

### ROI study and visual assessment

**Fig. 1:**
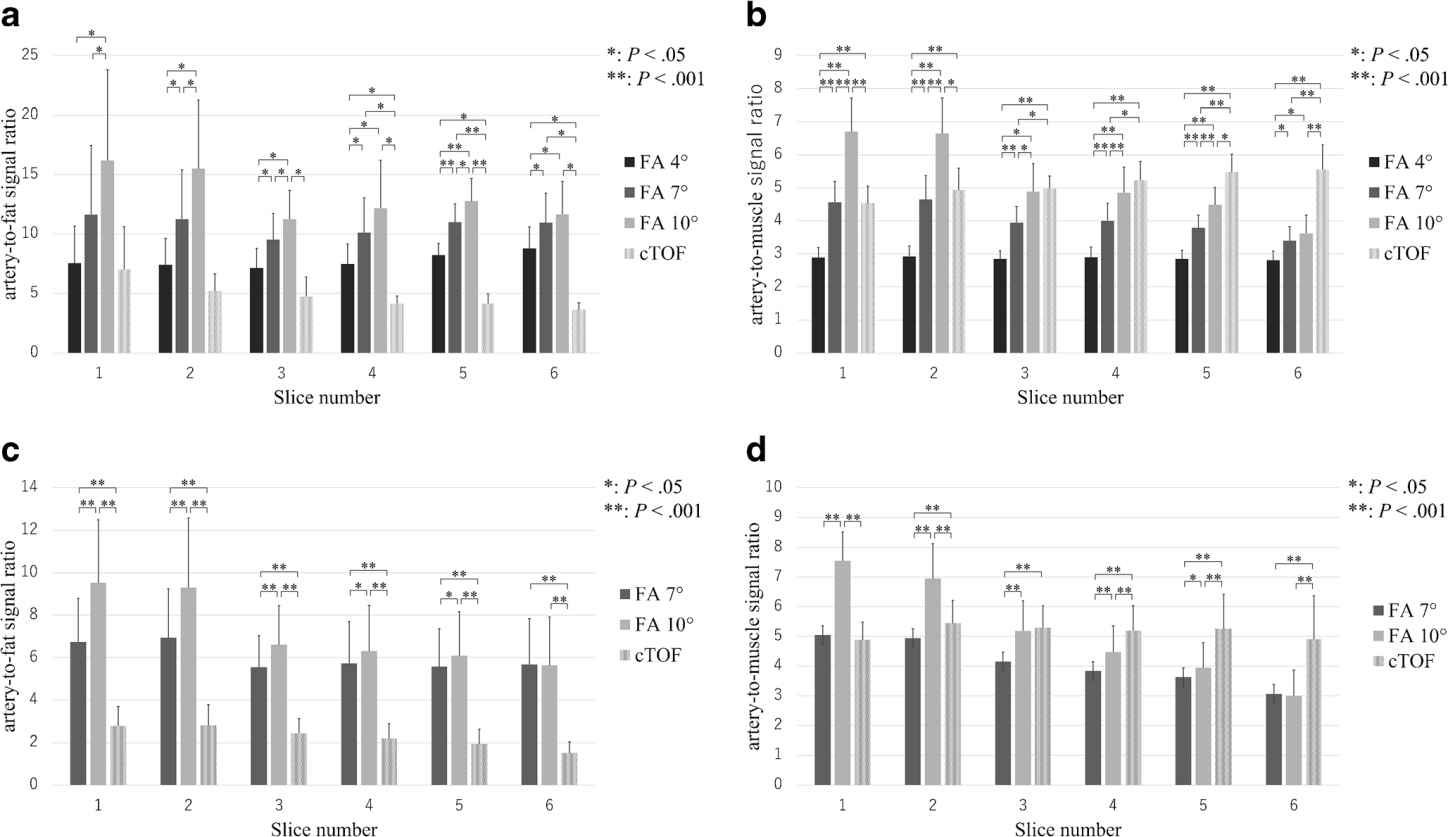
Artery-to-fat and artery-to-muscle signal intensity ratios of the volunteers (**a**, **b**) and of the patients (**c**, **d**). Mean signal ratio and standard deviation in six slices of the four sequences (LAVA MRA with FA 4°, FA 7°, and FA 10° and cTOF MRA) for volunteer data and three sequences (LAVA MRA with FA 7° and FA 10° and cTOF MRA) for patient data are shown as a bar graph. *P < 0.05; **P < 0.001

**Fig. 2:**
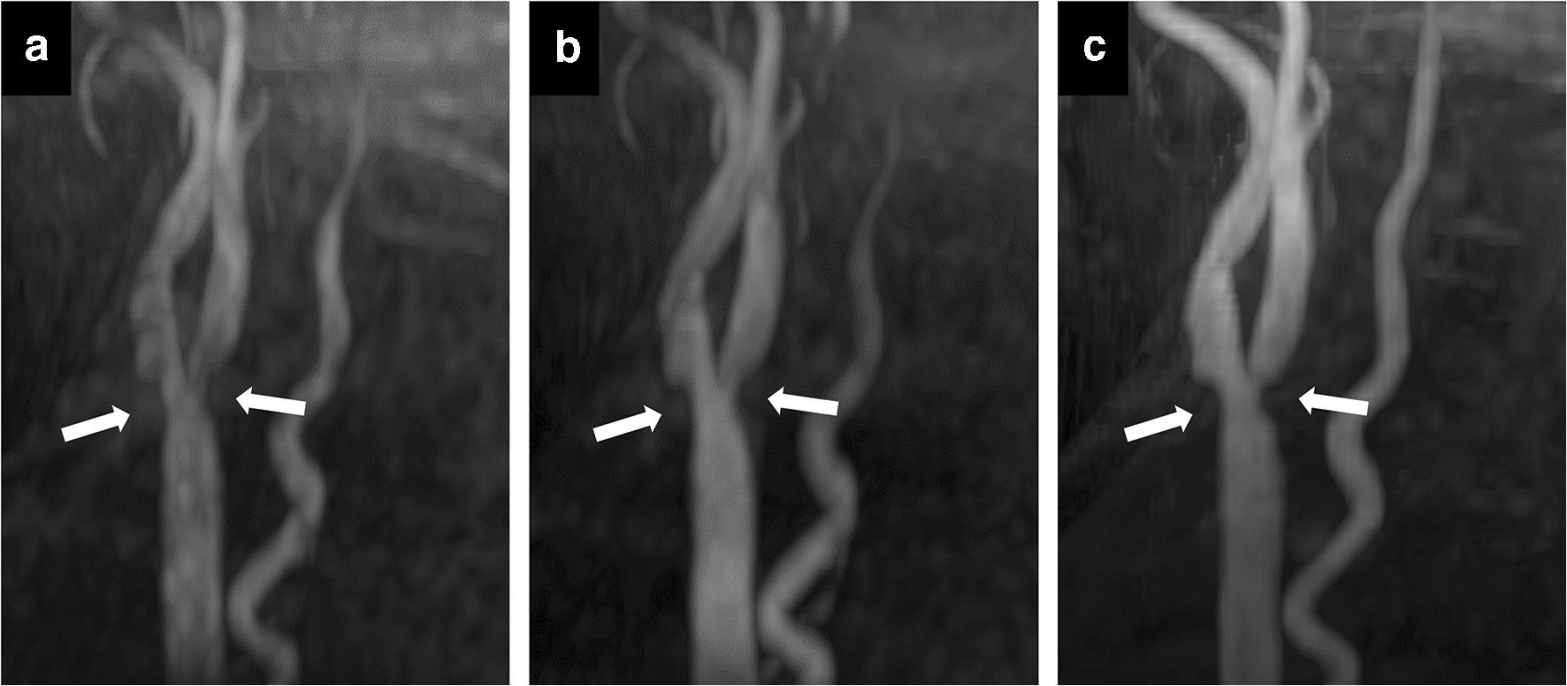
MIP images of a patient with cervical carotid stenosis. LAVA MRA with FA 7° (**a**) or FA 10° (**b**) can identify the shape of cervical carotid stenosis as effectively as cTOF MRA (**c**)

ROI study of the volunteers and patients are shown in Fig. 1 and the visual evaluation scores are shown in Table 2. Representative MIP images of each sequence in the patient are shown in Fig. 2.

**Table 2:** Visual evaluation scores of the patients

	FA 7°	FA 10°	cTOF
Image quality of MIP images	2.75 ± 0.44	2.81 ± 0.40	2.86 ± 0.48
Inhomogeneity due to the vortex flow	2.56 ± 0.95	2.34 ± 0.88	2.42 ± 0.89

Data are mean ± standard deviation. *FA 7°*, flip angle 7° (LAVA); *FA 10°*, flip angle 10° (LAVA); *cTOF*, conventional time-of-flight; *MIP*, maximum intensity projection

Repeated-measures ANOVA of SI_artery/fat_ and SI_artery/muscle_ of the volunteers (*F*_3,18_ > 5.06, *P* < 0.01; *F*_3,18_ > 53.6, *P* < 0.001, respectively) and that of the patients (*F*_2,38_ > 44.2, *P* < 0.001; *F*_2,38_ > 65.2, *P* < 0.001, respectively) found significant differences among the imaging sequences for all slices. In the volunteers, LAVA MRA with FA 10° exhibited a significantly larger (slices 3–6, Bonferroni corrected *P* < 0.05) or equivalent (slices 1 and 2) SI_artery/fat_ compared with cTOF MRA. LAVA MRA with FA 7° also exhibited a significantly larger (slices 4–6, *P* < 0.05) or equivalent (slices 1–3) signal ratio compared with cTOF MRA. LAVA MRA with FA 10° exhibited a significantly larger (slices 1 and 2, *P* < 0.001) or equivalent (slices 3 and 4) SI_artery/muscle_ in the common carotid and proximal internal carotid artery compared with cTOF MRA, respectively. LAVA MRA with FA 7° also exhibited an equivalent signal ratio in the common carotid artery compared with cTOF MRA (slices 1 and 2). By contrast, the signal ratio was significantly decreased in the distal part of the ICA (slices 3–6 for FA 7° and slices 5 and 6 for FA 10°, *P* < 0.05). LAVA MRA with FA 4° exhibited a significantly lower signal ratio compared with cTOF MRA (*P* < 0.001) in all slices. In the patients, LAVA MRA with FA 7° and 10° exhibited a significantly larger SI_artery/fat_ compared with cTOF MRA in all slices (*P* < 0.001). LAVA MRA with FA 10° exhibited a significantly larger (slices 1 and 2, *P* < 0.001) or equivalent (slice 3) SI_artery/muscle_ in the common carotid artery and the cervical carotid bifurcation compared with cTOF MRA. LAVA MRA with FA 7° also exhibited an equivalent SI_artery/muscle_ in the common carotid artery compared with cTOF MRA (slice 1). By contrast, a significantly decreased SI_artery/muscle_ was seen in the more distal part (slices 2–6 for FA 7° and slices 4–6 for FA 10°, *P* < 0.001).

In the visual assessment of the volunteers, there were significant differences among the four sequences about the scores of image quality based on the shape of carotid bifurcation (Friedman *χ*^2^ = 21.0, *P* < 0.001) and about the inhomogeneity scores due to the vortex flow (*χ*^2^ = 35.4, *P* < 0.001). The signal was significantly more homogeneous for FA 4° (*P* < 0.01) due to a low overall arterial signal, whereas it was not significantly different for FA 7°, FA 10°, and cTOF (*P* > 0.81, respectively). In the patients, there was no significant difference among the three sequences about the scores of image quality based on the shape of carotid bifurcation (*χ*^2^ = 2.33, *P* = 0.31; Nemenyi multiple comparisons, *P* > 0.77) and there was no significant difference among them about the scores of inhomogeneity due to the vortex flow (*χ*^2^ = 3.71, *P* = 0.15; Nemenyi multiple comparisons, *P* > 0.79). Weighted kappa coefficient was 0.73 in the image quality evaluation and 0.67 in the signal drop evaluation.

Comparison of cervical carotid stenosis rates showed no significant difference between LAVA MRA with FA 10° and cTOF MRA (0.47 ± 0.17 vs. 0.46 ± 0.21, *P* = 0.64). When the stenosis rate was classified as mild (< 50%), moderate (50–69%), or severe (≥ 70%), the results of the two sequences were completely consistent. In one case with severe stenosis, LAVA MRA was able to clearly identify the signal of the thin patent lumen (Supplement 3). When a signal defect caused by the jet-flow was recognized in the stenosis part, it was determined that the part was patent and the stenosis rate was measured (Supplement 4).

## Discussion

When the LAVA-Flex method in which the carotid MRA scanning is completed in only 29 s is adopted, the effect of time reduction is very large. Several reports have used parallel imaging or compressed sensing to reduce the time needed to complete carotid MRA [7, 8]. LAVA MRA can be acquired in a very short time compared with these methods.

Compared with cTOF MRA, arterial signal loss at the distal part of the ICA was greater with LAVA MRA, as expected. cTOF MRA applies a ramped excitation pulse to modulate the FA in order to prevent signal degradation at the distal end of the ICA [9, 10]. LAVA-Flex used in this study is a commercial sequence for the upper abdomen, so the improvement of image quality may be expected by creating a pulse sequence optimized for blood vessels, such as by introducing ramped excitation or by increasing the TR as long as the scan time can be kept short.

In this study, we set the cTOF MRA used in routine clinical practices as a reference standard. Non-CE MRA can overestimate the stenosis, mainly due to the vortex flow or the jet-flow at the site of severe stenosis. The lack of CE examinations can be a limitation of this study, but overestimation can also occur with CE MRA, which is sometimes worse than with non-CE MRA [11].

In conclusion, LAVA MRA can provide information similar to cTOF MRA for assessing the cervical carotid bifurcation while reducing scan time by one-fifth.

## Electronic supplementary material

ESM 1 (PNG 3582 kb) Regions of interest (ROIs) on the axial image of MR angiography.

High resolution image (TIF 2978 kb)

ESM 2 (PNG 9123 kb) MIP images of a healthy volunteer

High resolution image (TIF 10225 kb)

ESM 3 (PNG 6040 kb) Axial image of a patient with severe cervical carotid stenosis

High resolution image (TIF 5418 kb)

ESM 4 (PNG 7377 kb) A case with signal defect in the stenotic region

High resolution image (TIF 6557 kb)
