# Supplementary figures and images for: Accelerated acquisition of carotid MR angiography using 3D gradient-echo imaging with two-point Dixon
Source: Neuroradiology. 2020 May 18;62(10):1345–9. doi: 10.1007/s00234-020-02452-6 (PMC7479001; doi:10.1007/s00234-020-02452-6)

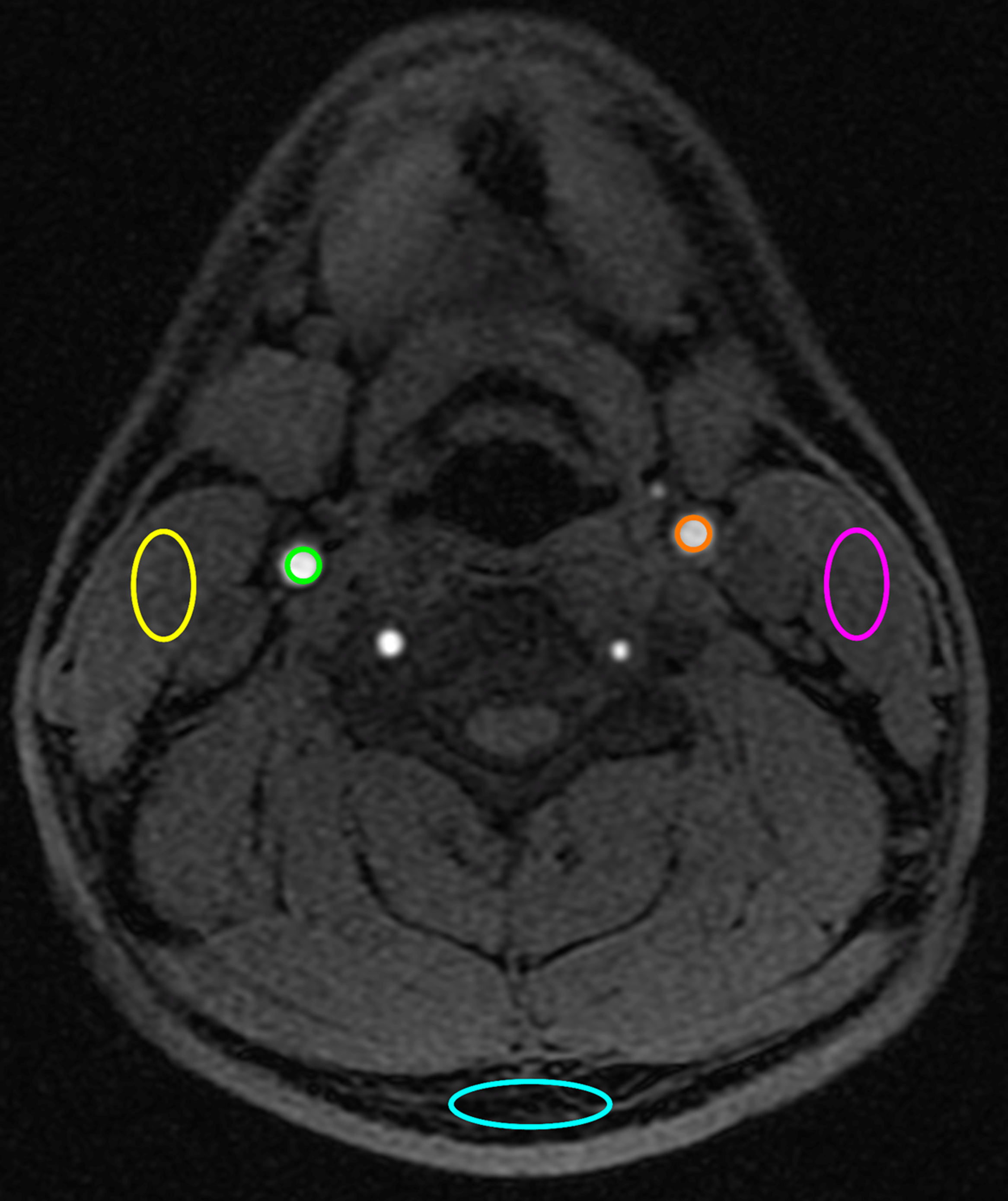

Supplement: Supplementary file 1 — (PNG 3582 kb) Regions of interest (ROIs) on the axial image of MR angiography. [file 234_2020_2452_Fig3_ESM.png]

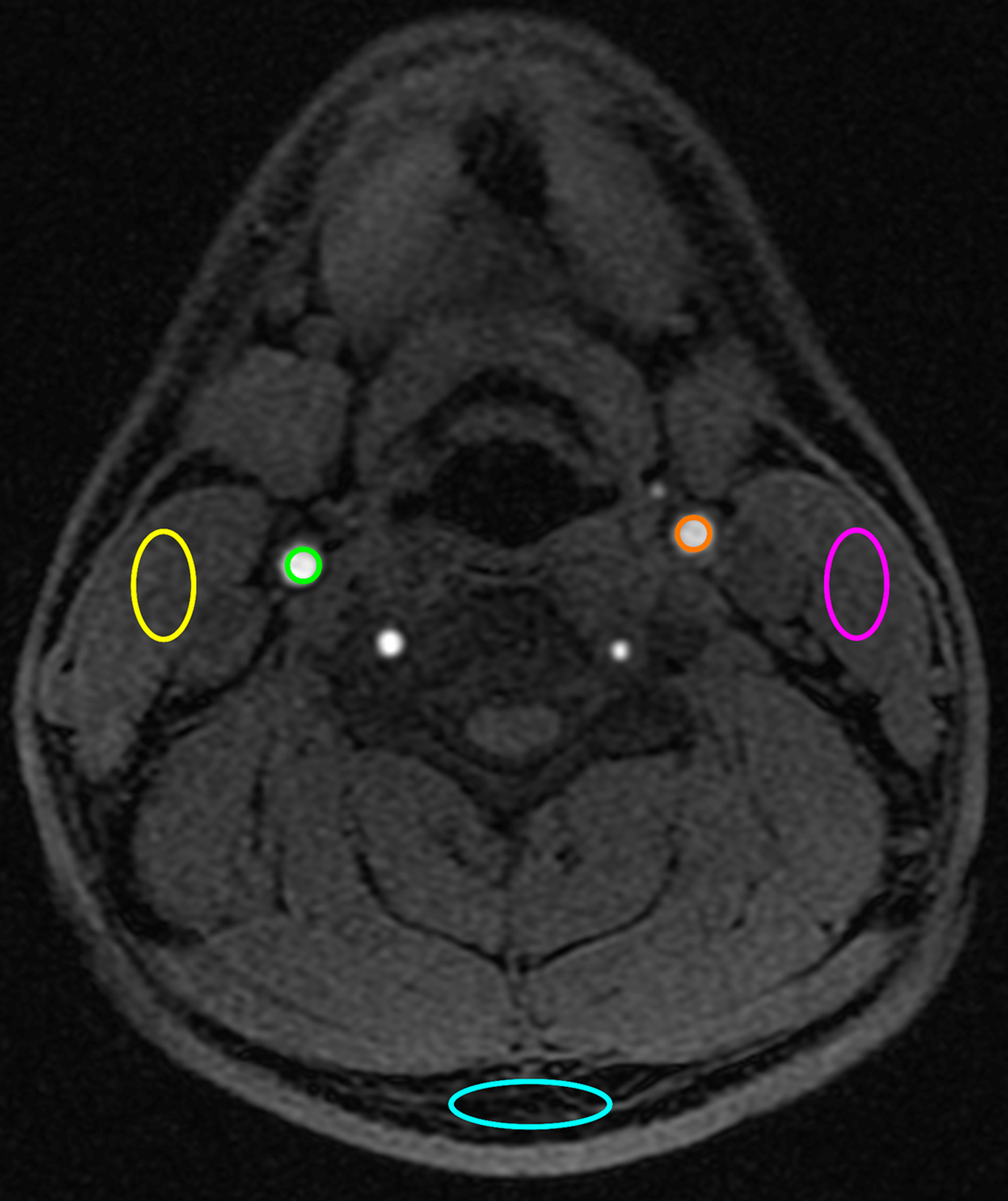

Supplement: Supplementary file 2 — High resolution image (TIF 2978 kb) [file 234_2020_2452_MOESM1_ESM.tif]

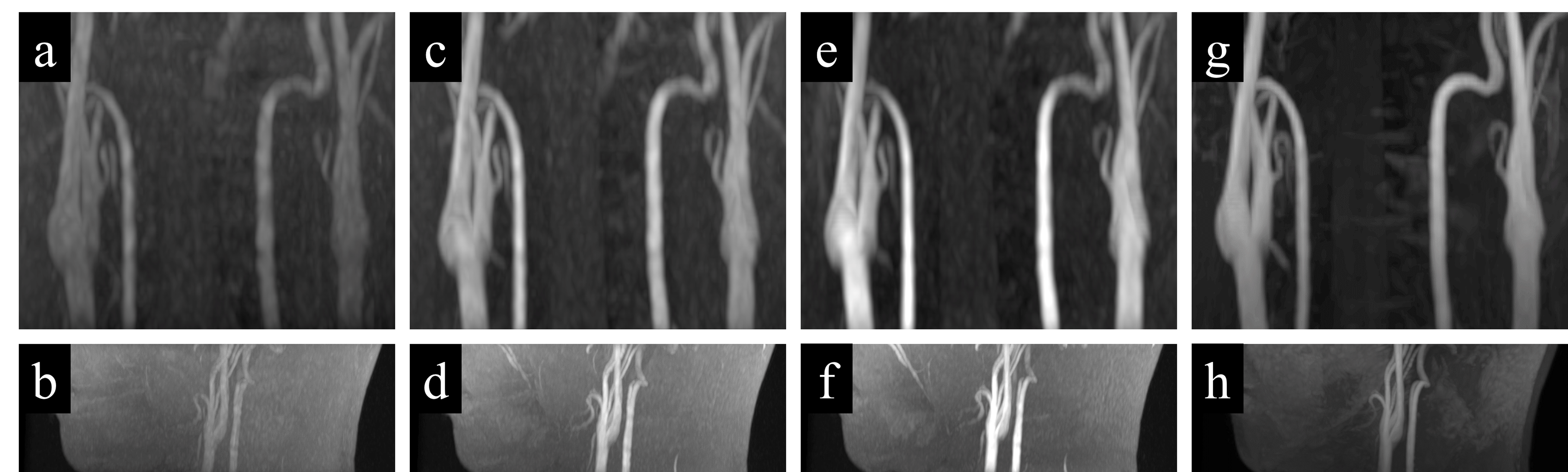

Supplement: Supplementary file 3 — (PNG 9123 kb) MIP images of a healthy volunteer [file 234_2020_2452_Fig4_ESM.png]

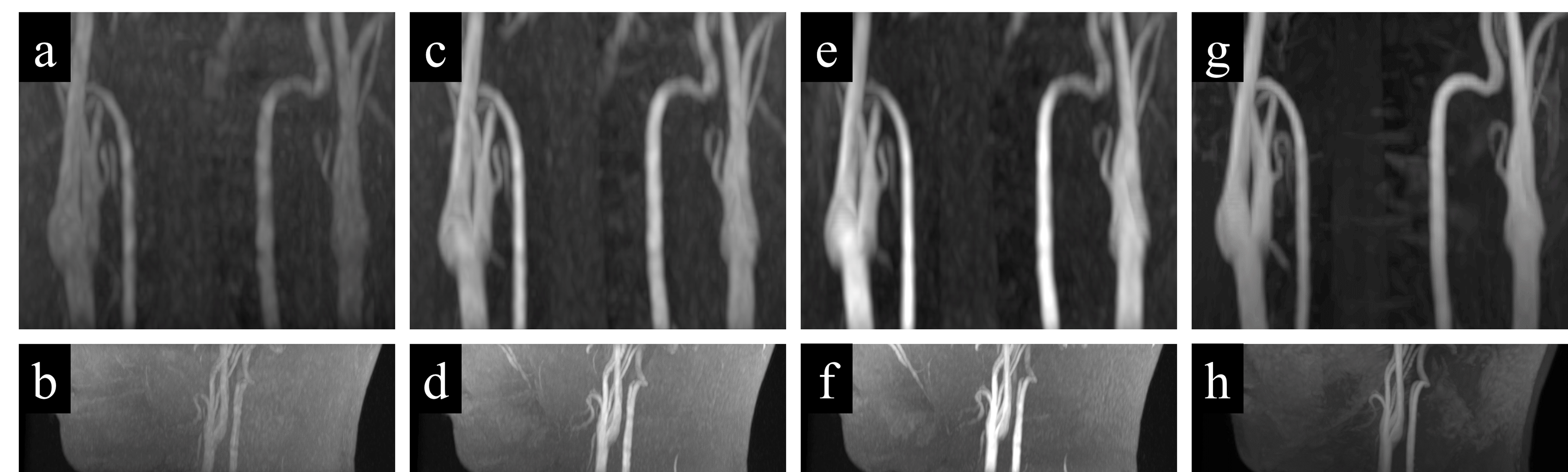

Supplement: Supplementary file 4 — High resolution image (TIF 10225 kb) [file 234_2020_2452_MOESM2_ESM.tif]

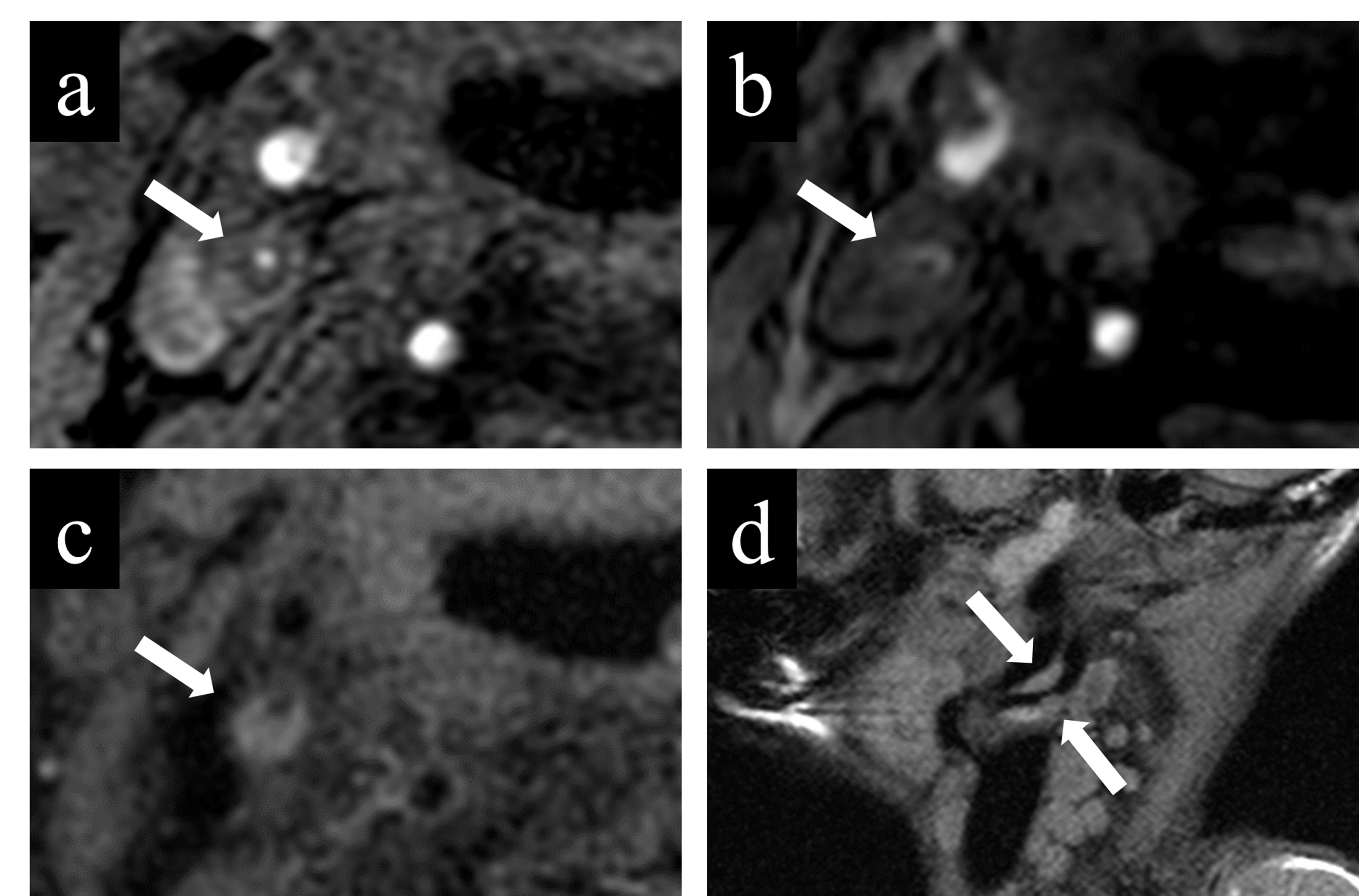

Supplement: Supplementary file 5 — (PNG 6040 kb) Axial image of a patient with severe cervical carotid stenosis [file 234_2020_2452_Fig5_ESM.png]

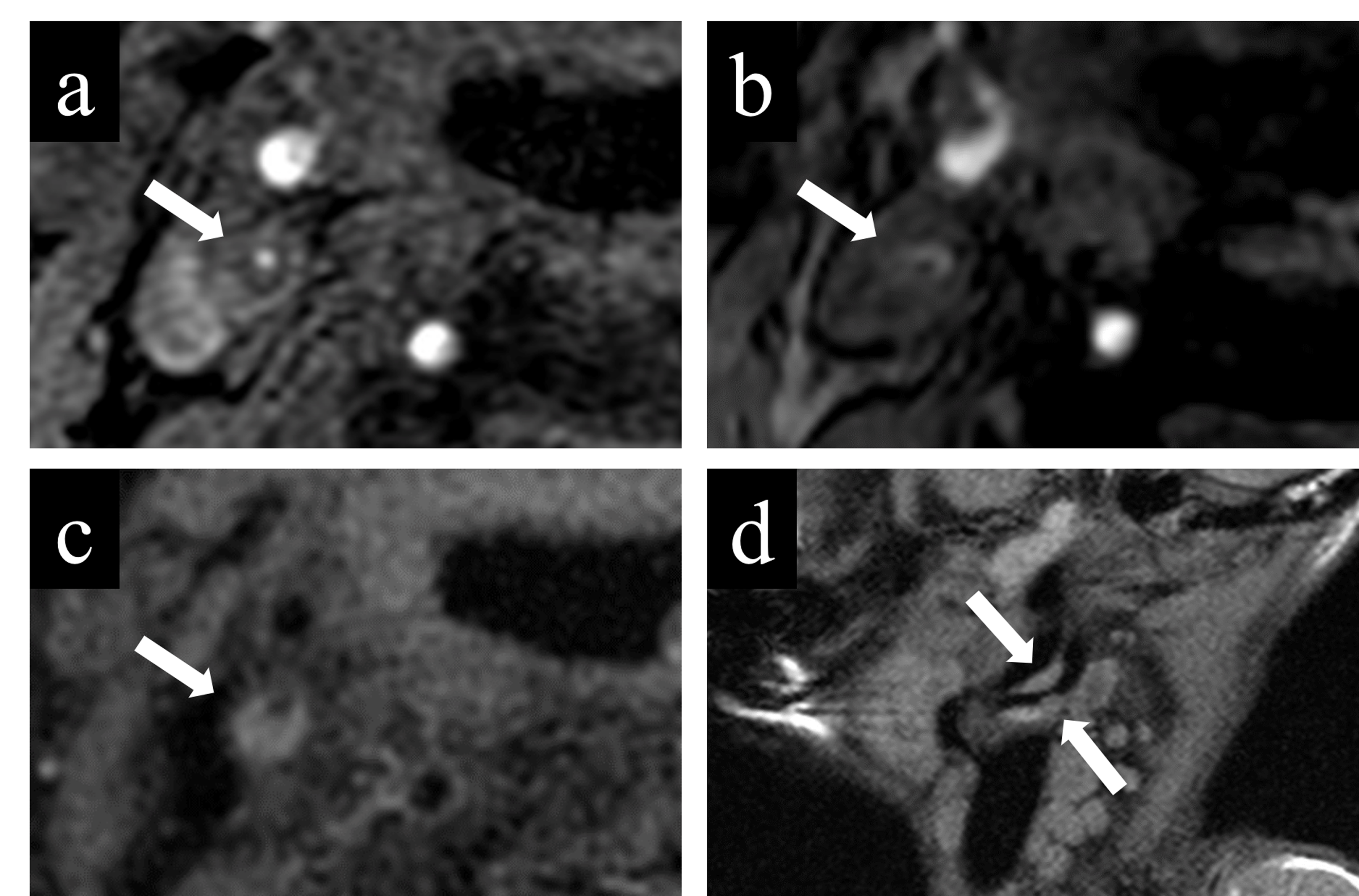

Supplement: Supplementary file 6 — High resolution image (TIF 5418 kb) [file 234_2020_2452_MOESM3_ESM.tif]

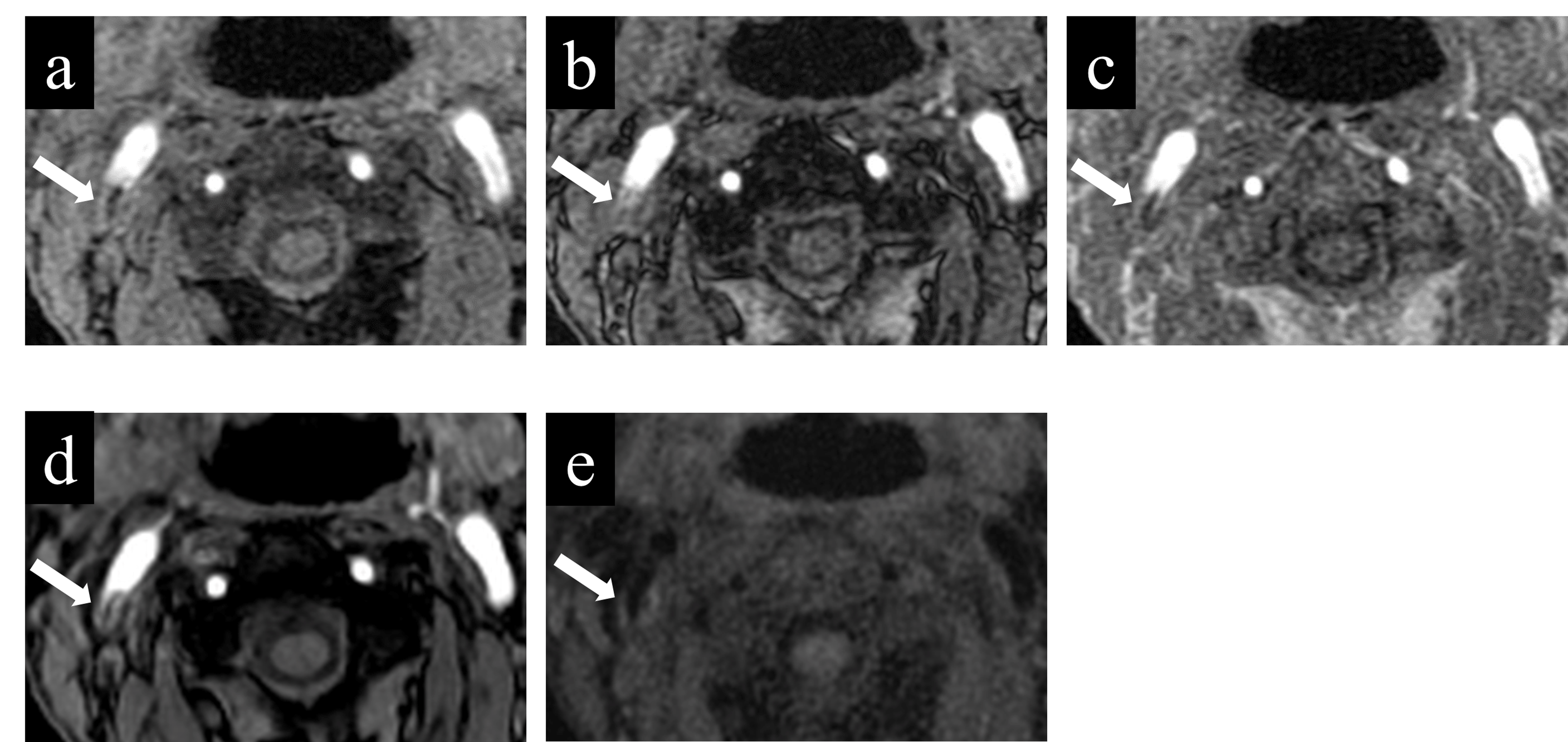

Supplement: Supplementary file 7 — (PNG 7377 kb) A case with signal defect in the stenotic region [file 234_2020_2452_Fig6_ESM.png]

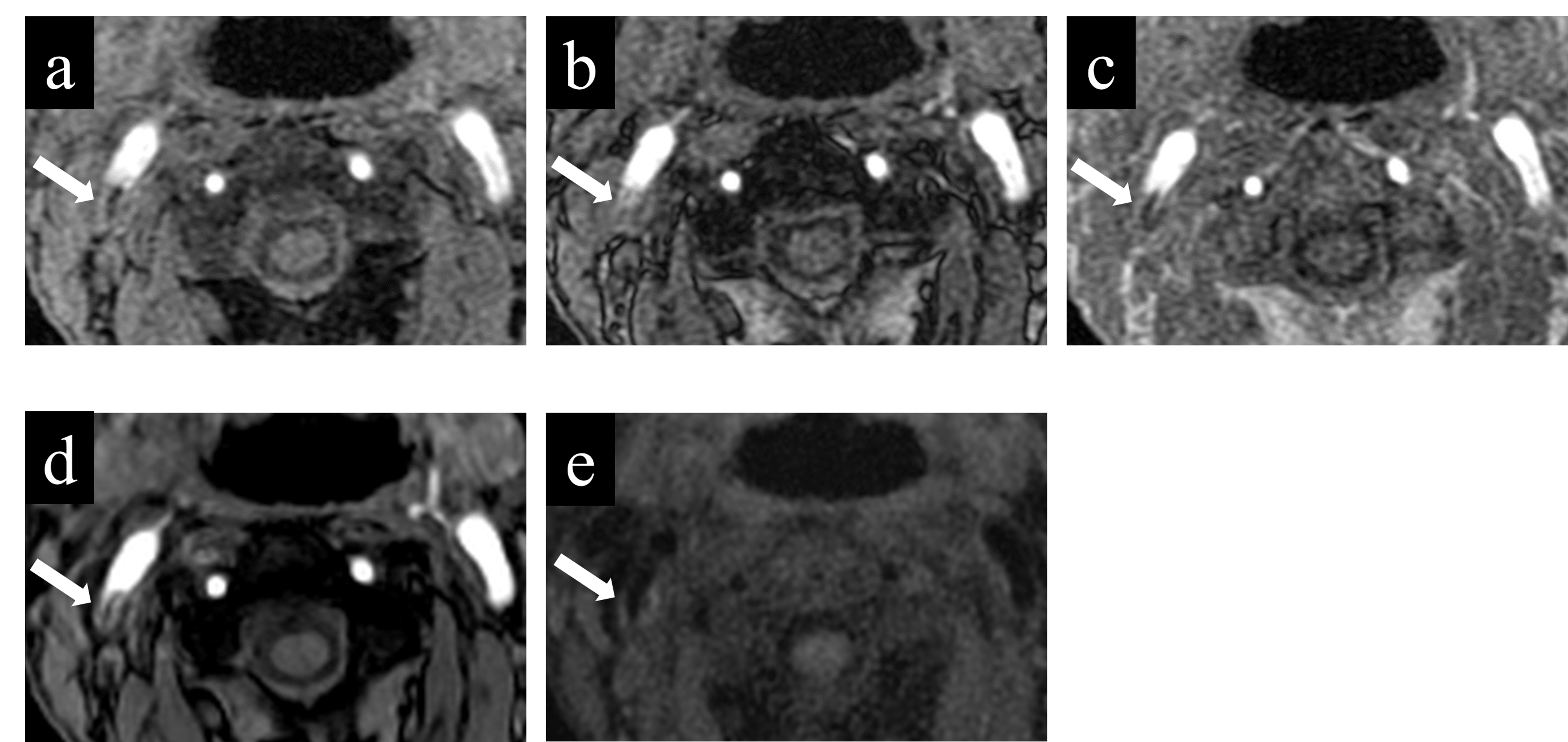

Supplement: Supplementary file 8 — High resolution image (TIF 6557 kb) [file 234_2020_2452_MOESM4_ESM.tif]
